# Supplementary material for: Structural Brain Imaging Predicts Individual-Level Task Activation Maps Using Deep Learning
Source: Front Neuroimaging. 2022 Apr 18;1:834883. doi: 10.3389/fnimg.2022.834883 (PMC10406267; doi:10.3389/fnimg.2022.834883)
Supplement: Supplementary file 1 [file Data_Sheet_1.pdf]

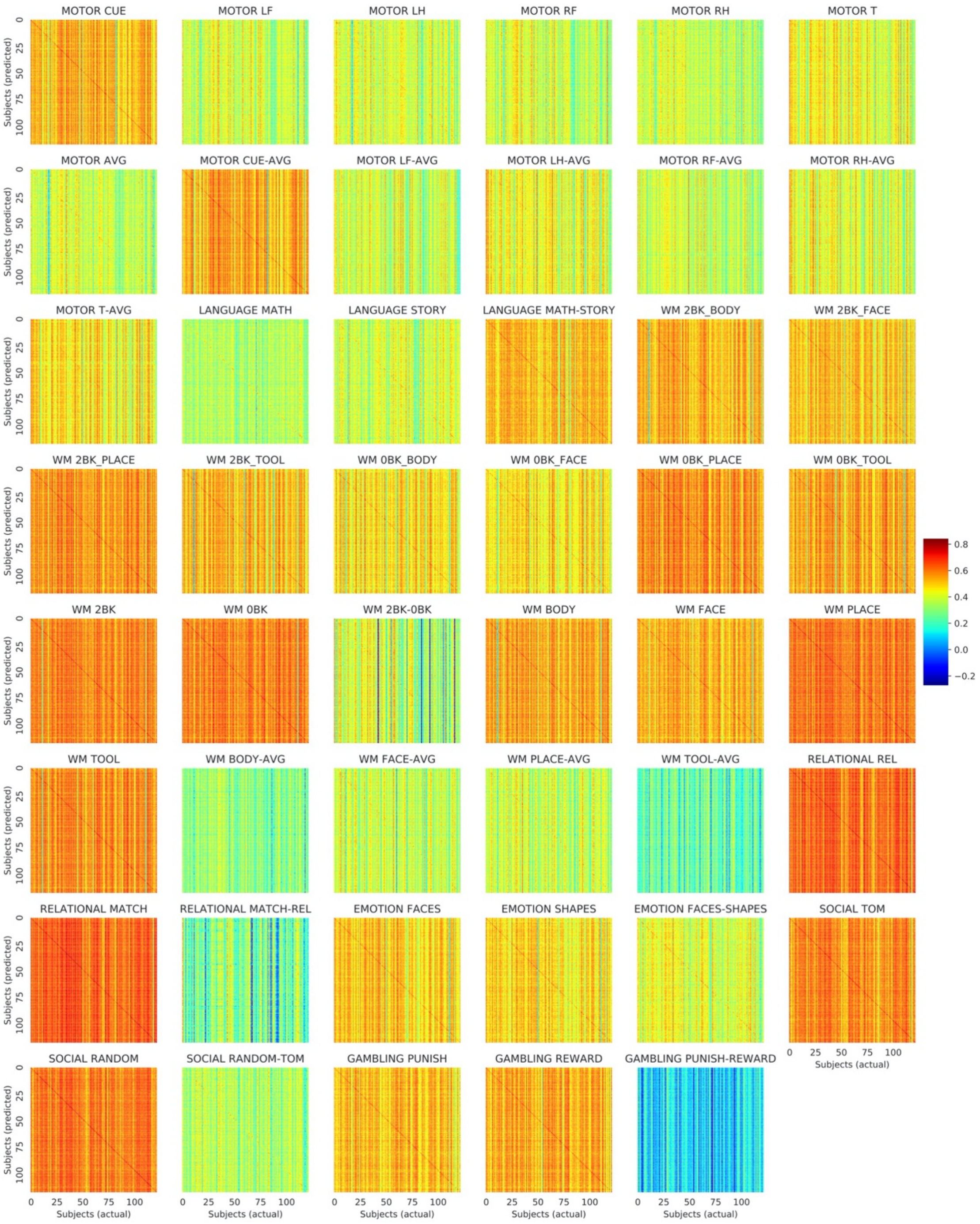

Figure S1 - Correlation matrices between the predicted task activation maps on the y-axis and the actual activation maps on the x-axis. A visible diagonal indicates that the predicted activation maps are more correlated with the respective actual activation map than with the activation maps of other subjects.

CUE=task cue; LF=left foot; LH=left hand; RF=right foot; RH=right hand; T=tongue; AVG=average; WM=Working Memory; 0BK=0 Back; 2BK=2 Back.

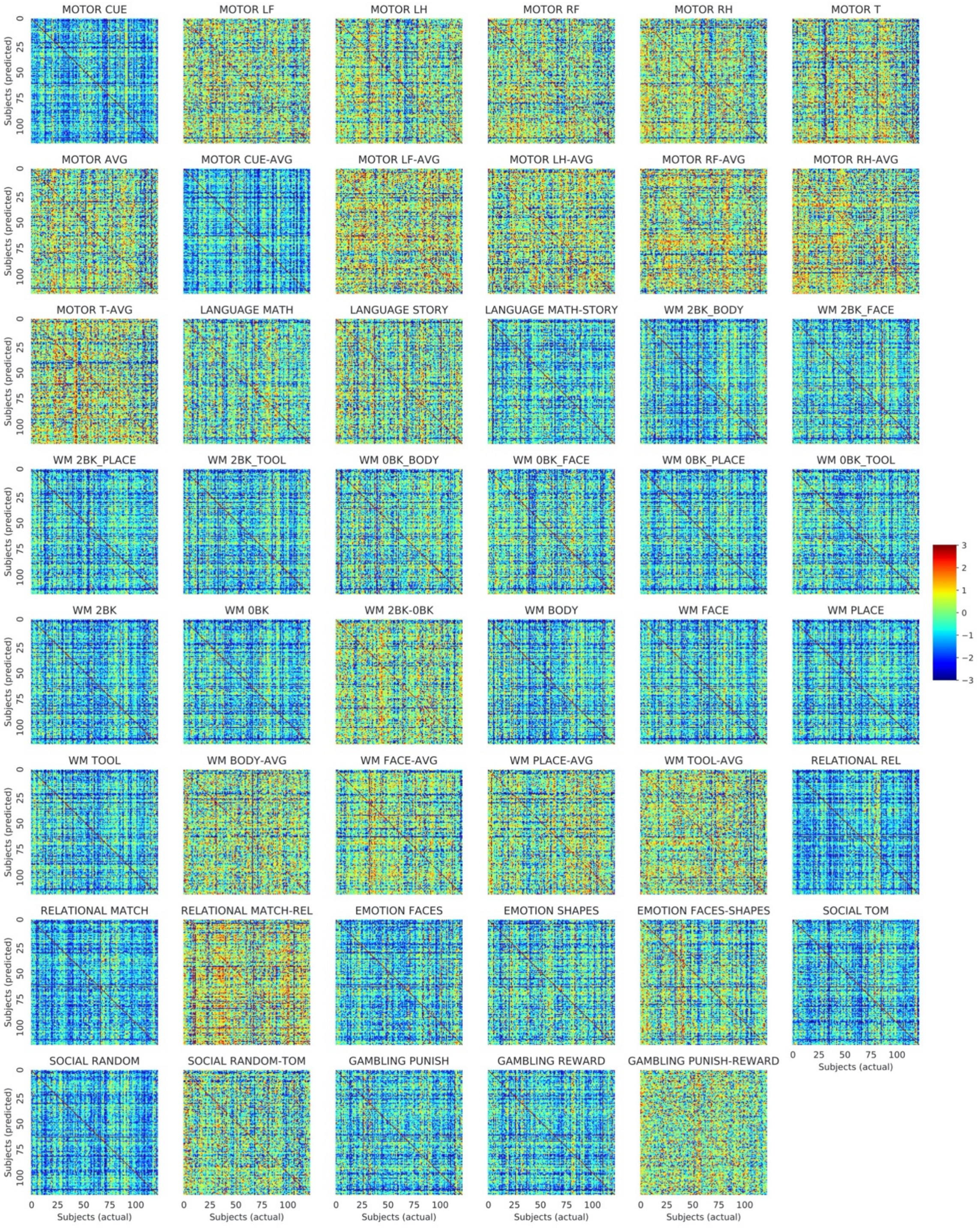

Figure S2 - Correlation matrices of the individual task maps that have been row and column normalized to account for the increased variability in the actual activation maps than in the predicted activation maps. The diagonal indicating the correlation between the predicted and actual maps is even more apparent after normalization.

CUE=task cue; LF=left foot; LH=left hand; RF=right foot; RH=right hand; T=tongue; AVG=average; WM=Working Memory; 0BK=0 Back; 2BK=2 Back.

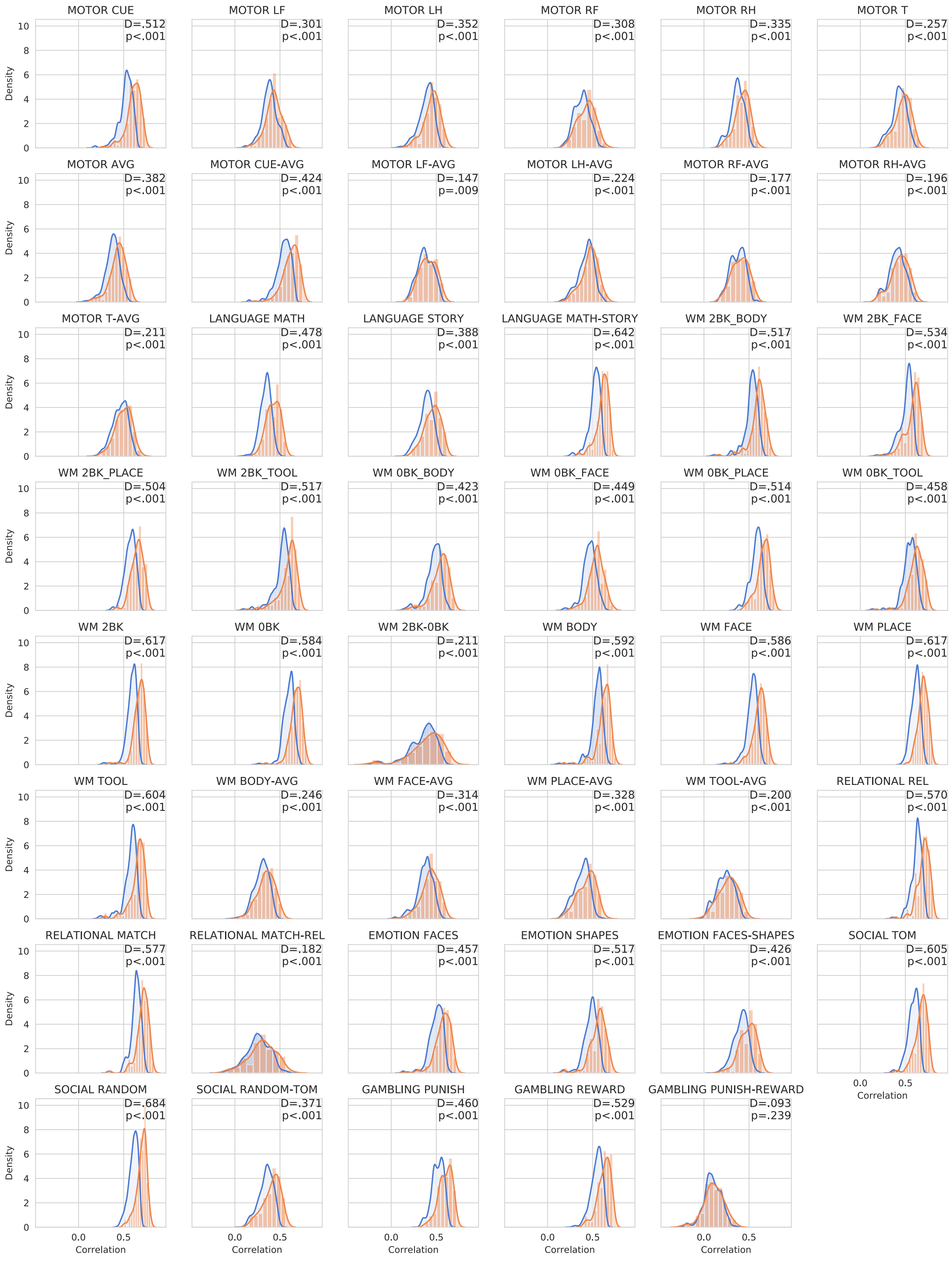

Figure S3 - Histogram comparing the correlation of the predicted individual motor and language task maps for a subject to the actual map for that subject in orange and the correlation between the predicted maps and the actual task activation maps for the other subjects in blue.

CUE=task cue; LF=left foot; LH=left hand; RF=right foot; RH=right hand; T=tongue; AVG=average; WM=Working Memory; 0BK=0 Back; 2BK=2 Back.

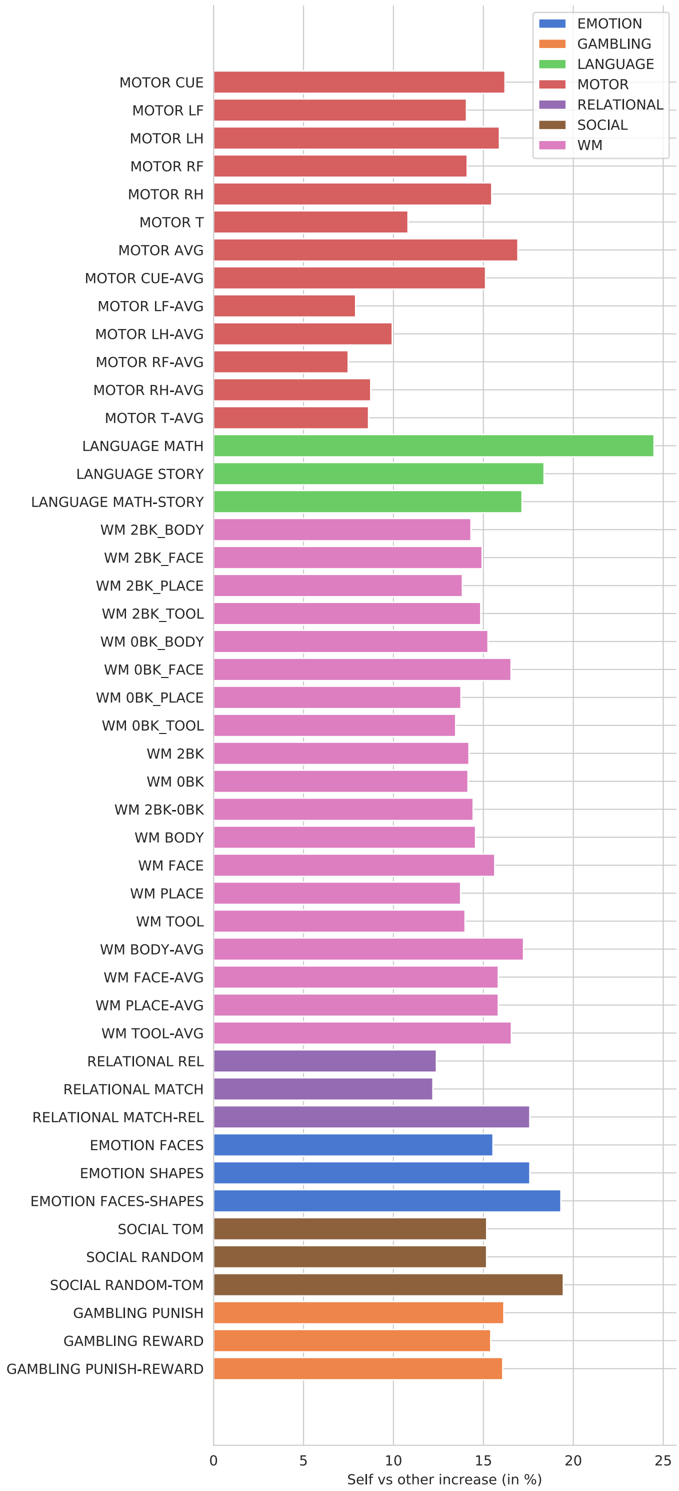

Figure S4 - **Self vs Other increase**. The difference between the average correlation between the predicted and actual maps (diagonal elements) and the average correlation between the predicted maps and the maps of all the other subjects (extra-diagonal elements) as a percentage relative to the average of the extra- diagonal elements. The positive values show that on average, the predictions match the actual maps (self) better than the average of the extra-diagonal elements (others).

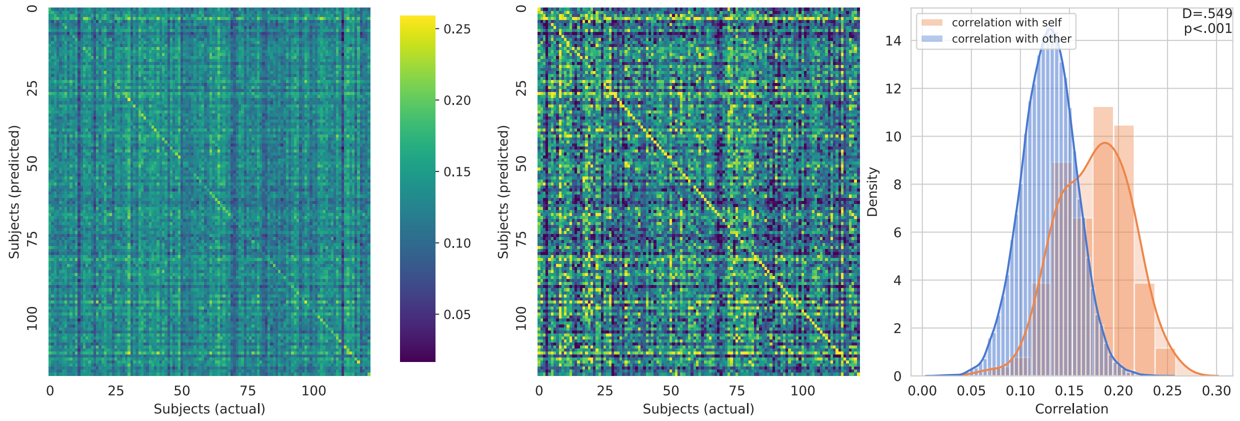

Figure S5 - (**A**) Partial correlation matrix for all tasks compared using the T1+T2 predictions as the covariates. The predicted maps (y-axis) were compared to the actual maps (x-axis) for all of the subjects. Even with using the T1+T2 predictions as the covariate, the visible diagonal indicates that the predicted maps were more correlated with their own actual maps than the maps of other subjects. (**B**) Row and column normalized correlation matrix to remove mean correlation. The normalized correlation matrix is heavily diagonal dominant. (**C**) Distribution of the diagonal elements of the (un-normalized) correlation matrix in orange and the extra-diagonal elements in blue visualized using a kernel density estimation and overlapping normalized histogram. A Kolmogorov-Smirnov test between the two distributions gives a highly significant difference $p < .001$. The differences in two distributions indicates that the microstructural features from the diffusion imaging are able to predict individual subject variation beyond that of the T1w and T2w anatomical imaging.

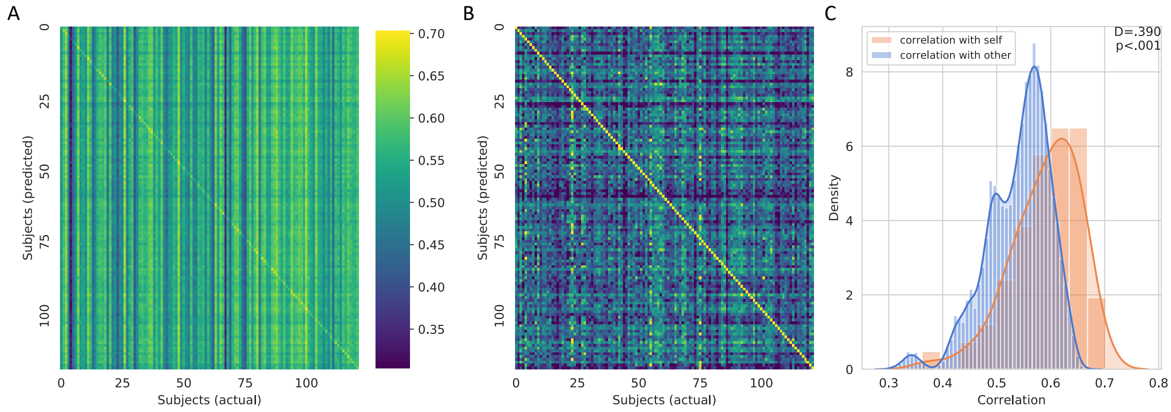

Figure S6 - (**A**) Correlation matrix for all tasks compared using MSMAll registered surface templates rather than the MSMSulc registered surface templates. The predicted maps (y-axis) were compared to the actual maps (x-axis) for all of the subjects. The MSMAll algorithm attempts to minimize individual subject variations in functional localization by using resting state fMRI and structural features for surface registration. Even with the MSMAll registration, the visible diagonal indicates that the predicted maps were more correlated with their own actual maps than the maps of other subjects. (**B**) Row and column normalized correlation matrix to remove mean correlation. The normalized correlation matrix is heavily diagonal dominant. (**C**) Distribution of the diagonal elements of the (un-normalized) correlation matrix in orange and the extra-diagonal elements in blue visualized using a kernel density estimation and overlapping normalized histogram. A Kolmogorov-Smirnov test between the two distributions gives a highly significant difference $p < .001$. This indicates individual variances in the task activation maps that the model is able to accurately predict are not solely the result of minor functional misalignments between subjects, as much of the variation was still predicted by the model even after the MSMAll surface templates corrected for the functional misalignment via registration.

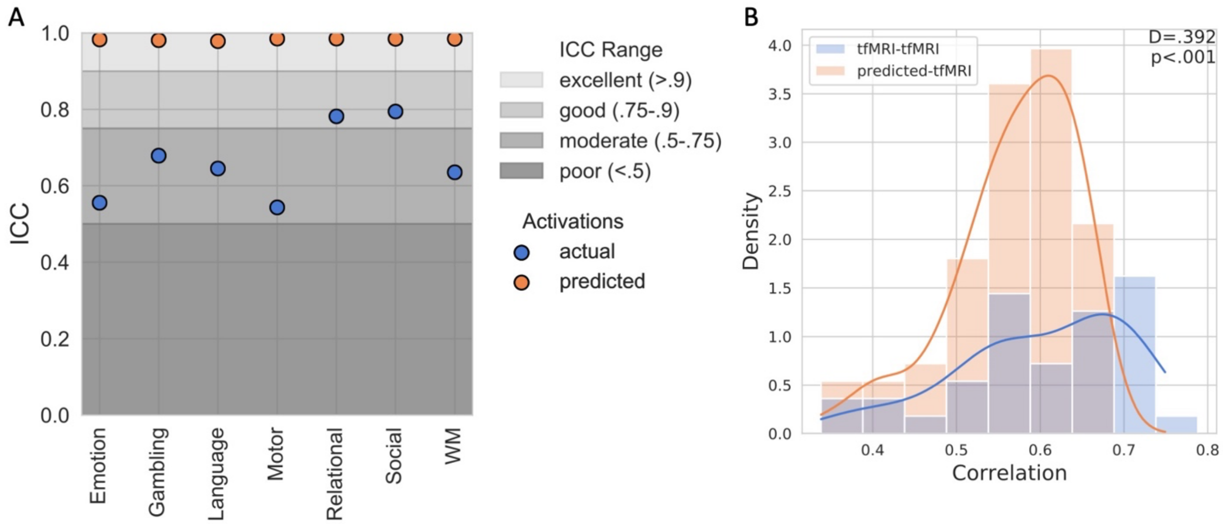

Figure S7 - **Test-retest analysis**. (**A**) Reliability of the predicted activation maps compared to that of the actual task-fMRI activation maps. Reliability was determined by assessing the intraclass correlation (ICC) of the maps over the entire cortex for subjects who were scanned and then rescanned four months later. The predicted maps had excellent ICC scores for all domains that were higher than the ICC scores of the actual task-fMRI maps. (**B**) Histogram and kernel density estimation for the test-retest correlation of actual task-fMRI maps to themselves in blue compared to the correlation of the predicted maps to the actual task-fMRI maps from the opposite test-retest session in orange. The predicted maps were not as correlated to the test-retest maps as the actual maps. Together these results show that the predicted maps were highly consistent but were not able to predict all the information that is captured by task fMRI.

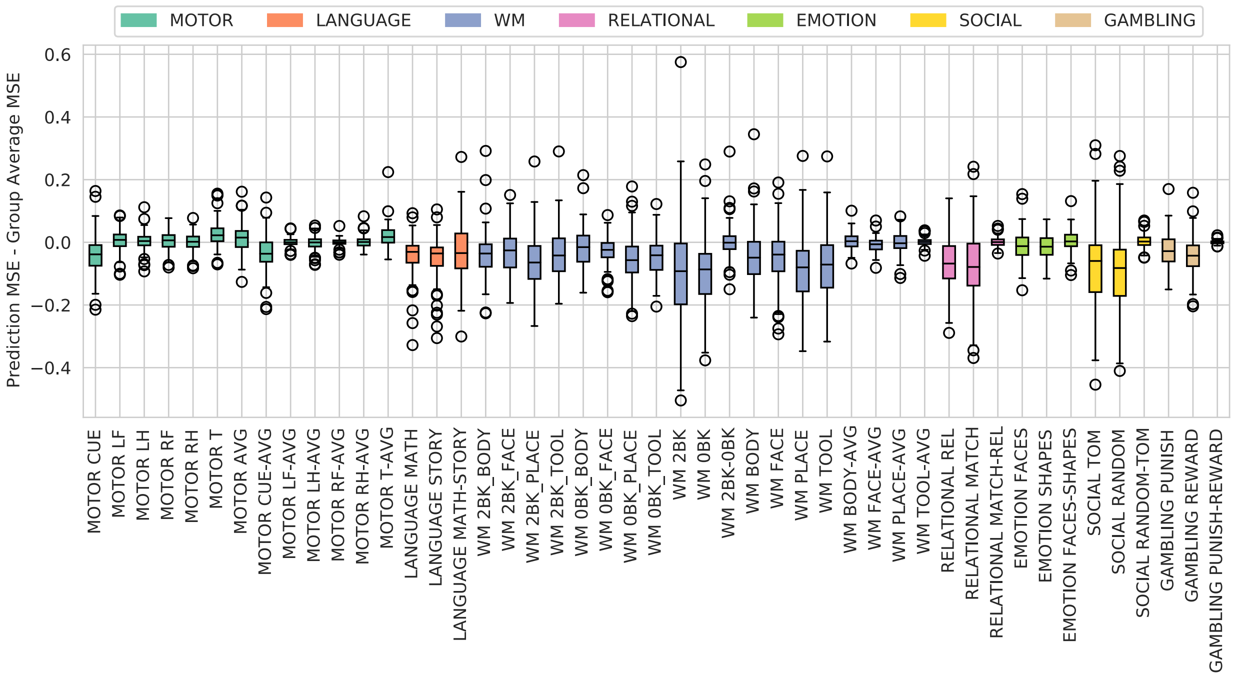

***Figure S8* - Prediction Squared Error Compared to Group Average Squared Error.** The activation volumes in the training group were non-linearly transformed to MNI space, averaged, and then compared to the activations in the test group. The predicted activation volumes were also transformed to MNI space and the mean squared error (MSE) of the prediction volumes for each of the test subjects was subtracted by MSE of the group average volumes to the test subject volumes. The predicted volumes showed lower MSE than the group average for many of the tasks, particularly those in the language, working memory, relational, and social domains. The average MSE for the predictions was 1.03 while the average MSE of the group average was 1.06.

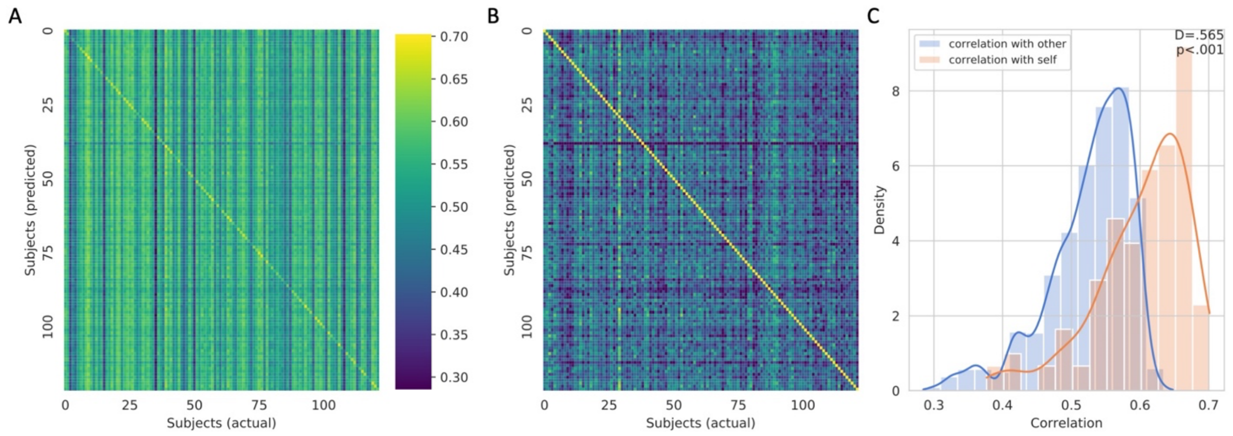

Figure S9 **- Reproduced results on separate randomized training splits.** An additional model was trained using randomized splits for the training, validation, and test sets. The test set was not completely random as the 42 subjects with retest data were included in the retest split along with the randomly assigned subjects. This figure shows that the results reported in this study can be reproduced on different sets of subjects.

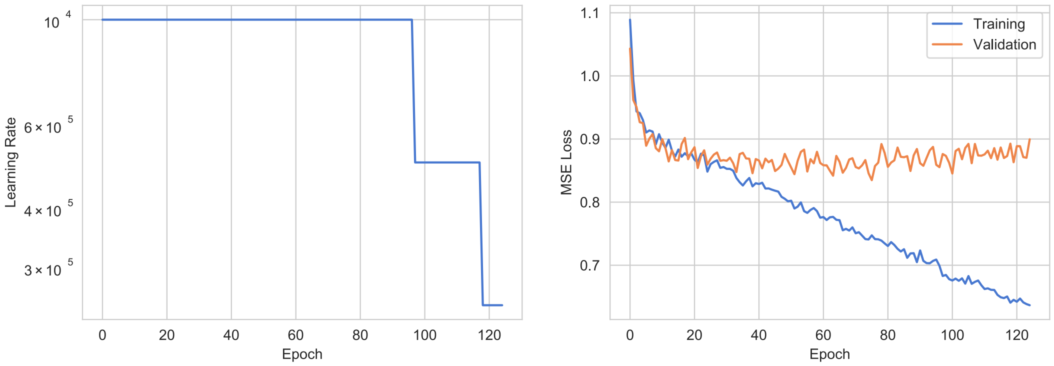

Figure S10 - Learning rate schedule (left) and training and validation loss (right) for the reproduced model whose results are shown in ***Figure S9***.

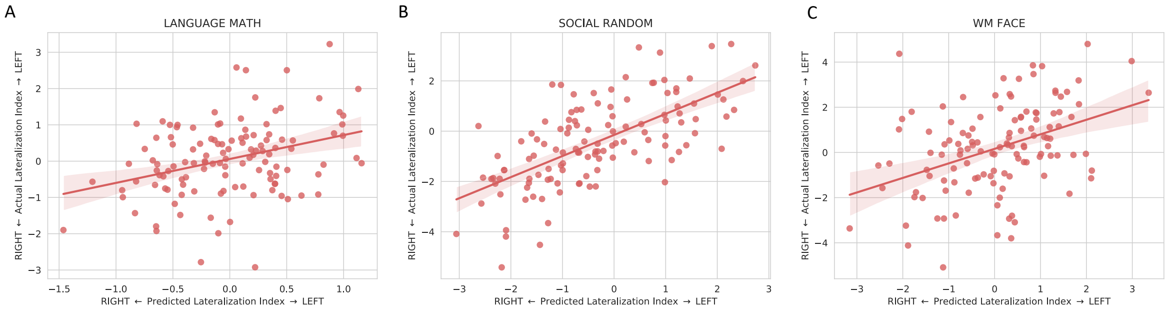

Figure S11* - Reproduced lateralization results on separate randomized training splits.** The model trained on separate randomized training splits as described in ***Figure S9 was able to reproduce lateralization results shown in the manuscript.

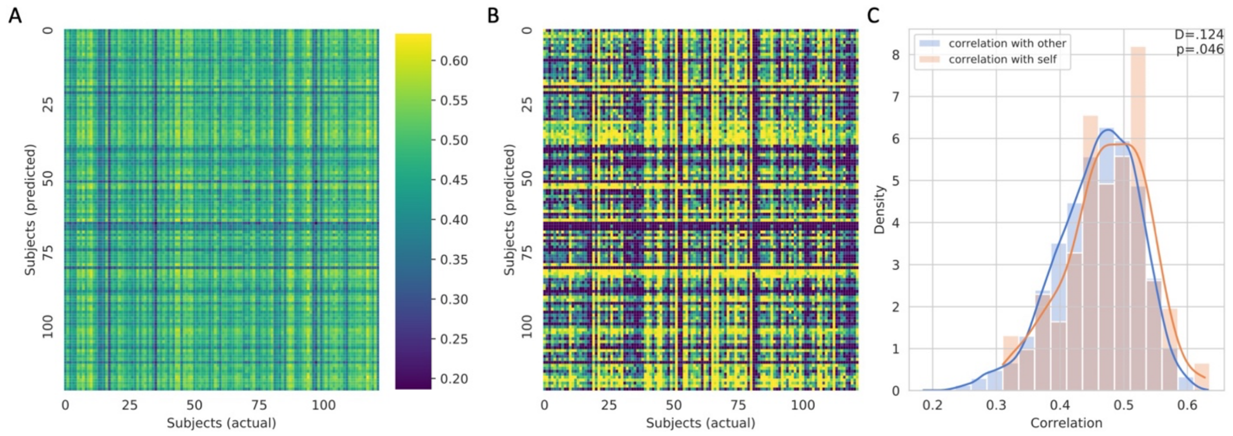

***Figure S12* - Correlation results of model trained on permuted input subjects**. A permutation test was conducted to ascertain the base level performance of a CNN on the output data. The input subject list was permuted prior to model training such that each input image was matched to an output image of a different subject. The model was then tested identically to the previous models reported in this study. Compared to the models trained on matching inputs and outputs, the model trained on permuted matchings does not display strong diagonal dominance in either the correlation matrix (**A**) nor the normalized correlation matrix (**B**). The histogram plots (**C**) show that the distribution of the "correlation with other" and "correlation with self" scores are highly overlapping. The p-value of the Kolmogorov-Smirnov test does fall below 0.05, but the corresponding D statistic for the test is 0.124 which is much less than the D statistic of 0.513 reported in this study.

Domain	Attributes	Activation Maps
Emotion	Shape matching compared to face matching with angry or fearful expressions	FACES, SHAPES, FACES-SHAPES
Gambling	Incentive processing, punishments, and rewards	PUNISH, REWARD, PUNISH-REWARD
Language	Auditory and phonological stories and arithmetic	STORY, MATH, MATH-STORY
Motor	Hand, foot, and tongue movements	CUE, LF, LH, RF, RH, T, AVG, CUE-AVG, LF-AVG, LH-AVG, RF-AVG, RH-AVG, T-AVG
Relational	Matching shapes and textures	REL, MATCH, MATCH-REL
Social	Random interactions compared to social interactions	TOM, RANDOM, RANDOM-TOM
Working Memory	N-back working memory, faces, places, tools, and body parts	2BK BODY, 2BK FACE, 2BK PLACE, 2BK TOOL, 0BK BODY, -0BK FACE, 0BK PLACE, 0BK TOOL, 2BK-0BK, BODY, FACE, PLACE, TOOL, BODY-AVG, FACE-AVG, PLACE-AVG, TOOL-AVG

Table S1 – Nomenclature and attributes for the task domains and activation maps included in this paper. The HCP uses the same nomenclature in their data releases and publications.

Domain	Contrast	Correlation	P-Value
LANGUAGE	MATH	0.45	1.47E-07*
LANGUAGE	STORY	0.28	1.82E-03*
LANGUAGE	MATH-STORY	0.31	4.16E-04*
WM	2BK_BODY	0.43	1.02E-06*
WM	2BK_FACE	0.48	2.21E-08*
WM	2BK_PLACE	0.48	3.22E-08*
WM	2BK_TOOL	0.41	3.49E-06*
WM	0BK_BODY	0.21	2.01E-02
WM	0BK_FACE	0.48	2.07E-08*
WM	0BK_PLACE	0.48	2.92E-08*
WM	0BK_TOOL	0.33	2.17E-04*
WM	2BK	0.48	2.42E-08*
WM	0BK	0.50	5.46E-09*
WM	2BK-0BK	0.19	3.49E-02
WM	BODY	0.39	6.84E-06*
WM	FACE	0.50	6.06E-09*
WM	PLACE	0.52	6.07E-10*
WM	TOOL	0.36	4.89E-05*
WM	BODY-AVG	-0.03	7.80E-01
WM	FACE-AVG	0.35	9.68E-05*
WM	PLACE-AVG	0.14	1.23E-01
WM	TOOL-AVG	0.40	6.13E-06*
SOCIAL	TOM	0.60	1.64E-13*
SOCIAL	RANDOM	0.63	5.11E-15*
SOCIAL	RANDOM-TOM	0.01	9.21E-01

*p<0.002

Table S2 - Pearson correlations between predicted and actual lateralization indices for select task domains. Task domains were selected to limit testing to those with expected variation between individuals. For significance testing, the α value was corrected for 25 multiple comparisons ($\alpha = \frac{0.05}{25} = 0.002$).

	MSE	MAE	IOU (z-score > 2)	IOU (z-score > 3)	IOU (z-score > 4)
**Prediction**	1.034	0.426	0.143	0.146	0.145
**All**	1.061	0.425	0.106	0.085	0.065
**Gender Matched**	1.061	0.425	0.107	0.086	0.066
**Age Matched**	1.064	0.426	0.106	0.085	0.065
**Gender + Age Matched**	1.068	0.426	0.106	0.085	0.066

***Table S3* - Comparison of scoring metrics for prediction and group average volumes.** Mean squared error (MSE), mean absolute error (MAE), and intersection over union (IOU) scores were compared for the predicted volumes as well as group average volumes in MNI space. The group average volumes consisted of a group average of all the training subjects as well as group average volumes of the training subjects that matched the test subject in either gender (M / F), age (22-25 / 25-30 / 31+), or both. The IOU scores were computed by thresholding the z-score or predicted z-score values of the volumes above 2, 3, or 4.

	ICC		
Domain	Original Predictions	Reproduced Predictions	tfMRI
Emotion	0.98	0.98	0.56
Gambling	0.98	0.98	0.68
Language	0.98	0.98	0.64
Motor	0.99	0.98	0.54
Relational	0.99	0.98	0.78
Social	0.98	0.98	0.79
WM	0.98	0.98	0.64

***Table S4* – Test-retest analysis with reproduced predictions**. Intraclass correlation (ICC) results for the original model as well as the model trained on random splits (see **Figure S9**). These results show that the reliability of the model can be reproduced on different sets of subjects from the dataset.
